# Normative Findings for Periocular Anthropometric Measurements among Chinese Young Adults in Hong Kong

**DOI:** 10.1155/2013/821428

**Published:** 2013-07-17

**Authors:** Yasas S. N. Jayaratne, Curtis K. Deutsch, Roger A. Zwahlen

**Affiliations:** ^1^Discipline of Oral & Maxillofacial Surgery, Faculty of Dentistry, The University of Hong Kong, Hong Kong; ^2^Eunice Kennedy Shriver Center, University of Massachusetts Medical School, Waltham, MA 02452, USA; ^3^Harvard Medical School, Boston, MA 02115, USA

## Abstract

Measurement of periocular structures is of value in several clinical specialties including ophthalmology, optometry, medical and clinical genetics, oculoplastic surgery, and traumatology. Therefore we aimed to determine the periocular anthropometric norms for Chinese young adults using a noninvasive 3D stereophotography system. Craniofacial images using the 3dMDface system were acquired for 103 Chinese subjects (51 males and 52 females) between the ages of 18 and 35 years. Anthropometric landmarks were identified on these digital images according to standard definitions, and linear distances between these landmarks were calculated. It was found that ocular measurements were significantly larger in Chinese males than females for intercanthal width, biocular width, and eye fissure lengths. No gender differences were found in the eye fissure height and the canthal index which ranged between 43 and 44. Both right and left eye fissure height-length ratios were significantly larger in females. This is the first study to employ 3D stereophotogrammetry to create a database of anthropometric normative data for periocular measurements. These data would be useful for clinical interpretation of periocular pathology and serve as reference values when planning aesthetic and posttraumatic surgical interventions.

## 1. Introduction

Measurement of periocular structures is of value in several clinical specialties including ophthalmology, optometry, medical and clinical genetics, oculoplastic surgery, and traumatology. The Online Mendelian Inheritance in Man (OMIM) database reveals almost 600 entries in which hypertelorism or telecanthus is a phenotypic feature [1]. Periocular dysmorphology is a cardinal feature for many genetic and teratogenic syndromes. For example, a shortened palpebral fissure width (i.e., between inner and outer canthi) is typically associated with fetal alcohol embryopathy/syndrome [2]. Periocular abnormalities can also arise through trauma, for example, traumatic telecanthus, which is often observed in nasoorbitoethmoid complex fractures. 

It is important to note that several interacting features such as epicanthic folds, flat nasal bridges, widely spaced eyebrows, or narrow palpebral fissures can give rise to the visual impression of hypertelorism [3, 4]. Thus accurate clinical measurements are needed for objective evaluation of periocular diseases as well as planning reconstructive surgery.

Traditionally anthropometric measurements in the periocular region have been performed using sliding calipers. However, measurements with such sharp instruments near the eye may pose some risk, especially when performing examination on very young or otherwise uncooperative patients. With the advent of commercial, high-resolution 3D stereophotogrammetry over the last decade, it has become possible to readily acquire craniofacial 3D photographs safely and expeditiously, and from these images it has been possible to obtain accurate and reliable measurements [5, 6]. These digital measurements are well suited to assessment of the periocular region. 

China has the world's largest population and residents with Chinese ancestry can be found in many countries. Using postmortem specimens, several anatomical differences between the Asian and Caucasian eyes have been demonstrated [7]. The first comprehensive clinical study on anthropometric norms of the Chinese faces was performed by Farkas et al. [8] in 1987. This study was conducted with manual anthropometry and the sample included Chinese subjects from Singapore aged 6, 12, and 18 years (*N* = 30 for each gender).

 In 2010, Wu et al. [9] investigated periocular anthropometric measurements of 53 male and 49 female Chinese undergraduate students from the Central South University in Hunan Province using traditional 2D photographs. However, such 2D measurement techniques rely on the assumption that the face is a flat object. Thus, the depth of periocular landmarks will confound these linear measurements. For example, the exocanthion and endocanthion are typically not located on the same line: the exocanthion is normally located posteroinferiorly to the endocanthion [10]. Therefore, for example, the eye fissure length measured on a 2D photograph may not be similar to that when measured using direct manual anthropometry. 3D photogrammetry overcomes this obstacle as it generates lifelike 3D facial images and at the same time provides a noninvasive tool to perform periocular measurements.

Individual patient's clinical anthropometric measurements are typically standardized by taking into account age, gender, and ethnicity, each of them being robust statistical covariates [11]. Normative data for 18–35 year olds are especially important, as persons of this age group are more likely to undergo oculoplastic procedures due to elective surgery or following trauma. The aim of this study was to provide a normative dataset of periocular anthropometric measurements using 3D imaging for Chinese young adults in Hong Kong.

## 2. Materials and Methods

This cross-sectional study was conducted using 3D photographs acquired from 103 Chinese subjects (51 males and 52 females) between 18 and 35 years of age. Sample subjects had no observable periocular pathology, and all patients with a history of oculoplastic surgery or orbital trauma were excluded. This study was approved by the Institutional Review Board of The University of Hong Kong/Hospital Authority Hong Kong West Cluster (Protocol no. UW 12-066). The sample was similar to the one used in our previous study [12]. 

**Figure 1:**
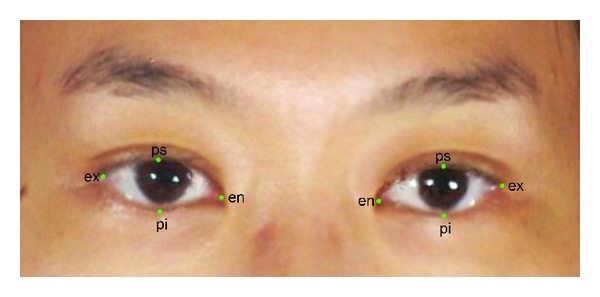
Anthropometric landmarks used in this study (en: endocanthion, ex: exocanthion, pi: palpebrale inferius, ps: palpebrale superius).

These 3D images were captured with the 3dMDface stereophotography system (3dMD, Atlanta, GA, USA) while the subjects were seated directly looking straight at the mirror to meet their own eyes (Figure 1). This imaging system consists of three pairs of synchronized cameras which simultaneously capture 6 images of the face (four monochromes and two colours) in less than 2 milliseconds. Based on a complex triangulation algorithm, these images are merged to generate a lifelike, accurate image of the face with the natural surface texture. Measurements using this system have been found to be reliable and valid [5, 6, 13].

**Figure 2:**
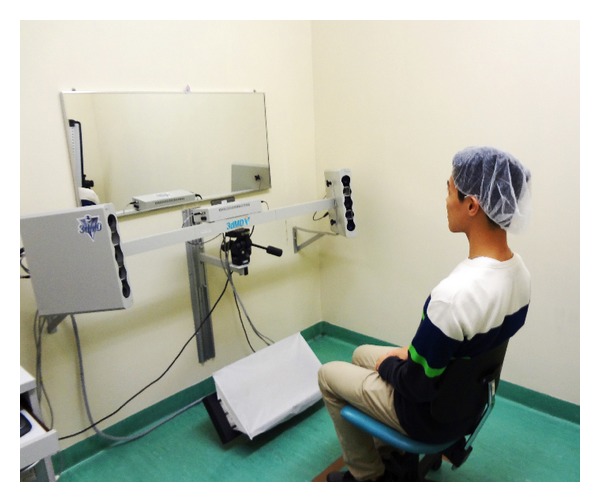
The 3dMDface stereophotography system used for image acquisition.

The acquired images were analyzed by a single investigator (YSNJ) using the *3dMDVultus* measurement software. Anthropometric landmarks (Figure 2) were first identified on each image according to standard definitions [14] (Table 1). The following linear measurements between these landmarks were automatically calculated by the software:

1. intercanthal width—distance between right and left endocanthions;
2. biocular width—distance between right and left exocanthions;
3. right and left eye fissure lengths—distance between endocanthion and exocanthion;
4. right and left eye fissure heights—distance between free edges of each eyelid (from palpebrale superius to palpebrale inferius).

The canthal index (Intercanthal width/Biocular width × 100) and eye fissure height-length ratio (ps-pi/ex-en × 100) were also computed.

Independent sample *t*-tests were conducted to compare the differences in the ocular measurements between both genders. 

## 3. Results

No statistically significant age differences were observed between the male (24.2 ± 3.03 years) and female (23.58 ± 3.91 years) groups (*P* = 0.372).

The means and standard deviations for each of the anthropometric eye measurements are presented in Table 2. Ocular measurements were significantly larger in Chinese males than females, except for the right and left eye fissure heights for which no significant sexual dimorphism was observed. No gender differences were found in the canthal index, which stood around 43-44. However, both right and left eye fissure height-length ratios were significantly larger in females. 

## 4. Discussion

As far as the authors are aware, this is the first study to utilize 3D stereophotography to create a database of anthropometric normative values for periocular measurements. Previous studies on periocular anthropometry were either based on manual anthropometry [8] or 2D photography [9]. 

Ethnicity- and gender-specific normative data for oculopalpebral anthropometric dimensions are invaluable for the clinical interpretation of ocular pathology, and they serve as reference points for aesthetic and posttraumatic surgical interventions. Satisfactory positioning of the medial canthal complex is crucial to achieve optimal intercanthal distance. The canthal index of 44 among Chinese underlines the fact that the traditional rule which stipulates that eyes length equals the intercanthal distance [12, 15] does not apply to this ethnic group. In addition, these normative data could be used in the detection and evaluation of genetic syndromes with periocular dysmorphology.

Our findings that men have larger dimensions than females in relation to intercanthal width (en-en), biocular width (ex-ex), and eye fissure length (ex-en) but no statistical differences between genders on the canthal index are in agreement with those of Wu et al. [9] on Chinese young adults. In contrast to this 2D photogrammetric study, we found no significance in measurements of right and left eye fissure heights between male and females. Wu et al. have used definitions they devised for palpebrae superioris (ps) and palpebrae inferioris (pi), rather than utilizing standard textbook definitions [10, 14]. They have defined ps as “*the midpoint of upper palpebral margin which was of the equal distance to En as to Ex*” and pi as the “*midpoint of lower palpebral margin, which was of the equal distance to En as to Ex*” [9]. In contrast, as listed in Table 1, we followed the definitions of Farkas [14]. For example, in the case of ps, the midpoint between En and Ex (according to Wu et al.) and highest point (according to Farkas) in the upper eyelid may not be the same and, consequently, the eye fissure height may not be the same. Therefore, Wu et al. may have found significant differences in measurements of right and left eye fissure height between males and females owing to imprecise identification of ps and pi. 

Considerable ethnic differences were apparent when we compared our findings to studies on young adults from other countries. The intercanthal width of Chinese was larger than their Turkish [16], Indian [17], Italian [18], North American Caucasian [19], or African-American [20] counterparts. The biocular width was comparable to other ethnic groups but considerably larger than Turkish young adults. The Chinese had shorter eye fissures than Italians, North American Caucasians, or African-Americans. 

One limitation of this study was the acquisition of subjects primarily located in Hong Kong. Recruitment of participants from other parts of China would have been a labor- and cost-intensive task. Nevertheless, as Hong Kong Chinese belong to the common “Han” ethnicity, the largest ethnic group in China and indeed, in the world [21, 22], this group constitutes a practical choice for initial documentation of Chinese craniofacial morphology. The normative values established in our study may also be useful even when evaluating Chinese living abroad, where the Han ethnicity is also overrepresented among individuals self-identified as Chinese [23].

## 5. Conclusion

This study establishes normative anthropometric periocular measurements for Chinese young adults in Hong Kong. Intercanthal width, biocular width, and eye fissure lengths were significantly larger in Chinese males than females, but the eye fissure height did not differ between genders. The normative anthropometric data presented in this study would be useful for clinical interpretation of periocular pathology and serve as reference values when planning aesthetic and posttraumatic surgical interventions.

## Figures and Tables

**Table 1:** Definitions of Anthropometric landmarks used in this study.

Landmark	Abbreviation	Definition [14]
Exocanthion	ex	The soft tissue point located at the outer commissure of each eye fissure
Endocanthion	en	The soft tissue point located at the inner commissure of each eye fissure
Palpebrale superius	ps	The highest point in the mid portion of the free margin of each upper eyelid
Palpebrale inferius	pi	The lowest point in the mid portion of the free margin of each lower eyelid

**Table 2:** Normative eye measurements.

Anthropometric parameter	Gender	*N*	Mean	SD	*P*-value
Intercanthal width (en-en)	Male	51	40.61	4.91	0.003
	Female	52	38.27	2.61	
Biocular width (ex-ex)	Male	51	93.00	5.56	<0.001
	Female	52	88.39	3.74	
Right eye fissure length (ex-en)	Male	51	27.64	1.67	<0.001
	Female	52	26.04	1.83	
Right eye fissure height (ps-pi)	Male	51	11.55	1.05	0.086
	Female	52	11.94	1.23	
Left eye fissure length (ex-en)	Male	51	27.07	1.74	<0.001
	Female	52	25.37	1.43	
Left eye fissure height (ps-pi)	Male	51	11.64	1.71	0.993
	Female	52	11.64	1.30	
Canthal Index (en-en/ex-ex × 100)	Male	51	43.95	8.23	0.580
	Female	52	43.29	2.35	
Right eye fissure height-length ratio (ps-pi/ex-en × 100)	Male	51	41.84	3.69	<0.001
	Female	52	45.89	4.05	
Left eye fissure height-length ratio (ps-pi/ex-en × 100)	Male	51	43.06	6.07	<0.001
	Female	52	45.88	4.42	
